# Niclosamide as a Repurposing Drug against *Corynebacterium striatum* Multidrug-Resistant Infections

**DOI:** 10.3390/antibiotics11050651

**Published:** 2022-05-12

**Authors:** Veronica Folliero, Federica Dell’Annunziata, Emanuela Roscetto, Marcella Cammarota, Anna De Filippis, Chiara Schiraldi, Maria Rosaria Catania, Vincenzo Casolaro, Alessandro Perrella, Massimiliano Galdiero, Gianluigi Franci

**Affiliations:** 1 Department of Experimental Medicine, University of Campania “Luigi Vanvitelli”, 80138 Naples, Italy; veronica.folliero@unicampania.it (V.F.); federica.dellannunziata@unicampania.it (F.D.); marcella.cammarota@unicampania.it (M.C.); anna.defilippis@unicampania.it (A.D.F.); chiara.schiraldi@unicampania.it (C.S.); 2 Department of Molecular Medicine and Medical Biotechnology, University of Naples Federico II, 80131 Naples, Italy; emanuela.roscetto@unina.it (E.R.); mariarosaria.catania@unina.it (M.R.C.); 3 Department of Medicine Surgery and Dentistry, University of Salerno, Baronissi, 84081 Salerno, Italy; vcasolaro@unisa.it; 4 Division Emerging Infectious Disease and High Contagiousness, Hospital D Cotugno, 80131 Naples, Italy; alessandro.perrella@ospedalideicolli.it; 5 Clinical Pathology and Microbiology Unit, San Giovanni di Dio e Ruggi D’Aragona University Hospital, 84126 Salerno, Italy

**Keywords:** *Corynebacterium striatum*, drug-repurposing, multidrug-resistant pathogen

## Abstract

*Corynebacterium striatum* (*C. striatum*) is an emerging multidrug-resistant (MDR) pathogen associated with nosocomial infections. In this scenario, we screened the antimicrobial activity of the anthelmintic drugs doramectin, moxidectin, selamectin and niclosamide against 20 *C. striatum* MDR clinical isolates. Among these, niclosamide was the best performing drug against *C. striatum*. Niclosamide cytotoxicity was evaluated by a 3-(4,5-dimethylthiazol-2-yl)-2,5-diphenyl tetrazolium bromide (MTT) assay on immortalized human keratinocyte cells (HaCaT). After 20 h of treatment, the recorded 50% cytotoxic concentration (CC_50_) was 2.56 μg/mL. The antibacterial efficacy was determined via disc diffusion, broth microdilution method and time-killing. Against *C. striatum*, niclosamide induced a growth inhibitory area of 22 mm and the minimum inhibitory concentration that inhibits 90% of bacteria (MIC_90_) was 0.39 μg/mL, exhibiting bactericidal action. The biofilm biomass eradicating action was investigated through crystal violet (CV), MTT and confocal laser scanning microscopy (CLSM). Niclosamide affected the biofilm viability in a dose-dependent manner and degraded biomass by 55 and 49% at 0.39 μg/mL and 0.19 μg/mL. CLSM images confirmed the biofilm biomass degradation, showing a drastic reduction in cell viability. This study could promote the drug-repurposing of the anthelmintic FDA-approved niclosamide as a therapeutic agent to counteract the *C. striatum* MDR infections.

## 1. Introduction

Healthcare-associated infections (HCAIs) represent a serious threat to hospitalized patients, with worrying economic and managerial effects on public health [1]. The United States Centers for Disease Control and Prevention (CDC) estimates that about 1.7 million hospitalized patients develop nosocomial infections annually, with a mortality rate exceeding 6% [2]. In the last decade, an emerging multidrug-resistant nosocomial pathogen in hospitalized patients has been reconducted to the genus Corynebacterium [3,4].

Corynebacteria are non-spore-forming bacteria with considerable pleomorphism, from club-shaped to long slender bacilli [2]. These bacteria are widely spread in the environment and constitute part of the human and animal skin microbiota [5]. *Corynebacterium striatum* (*C. striatum*) is increasingly causing serious infections in immunocompetent and immunocompromised patients [5]. These bacteria are involved in bacteremia, endocarditis, meningitis, vaginitis, respiratory and urinary tract infections, as well as infections of the skin and eye wounds [6]. *C. striatum* has only recently been recognized as a nosocomial pathogen and not simply a contaminant, due to the increase of its multi-drug resistance (MDR) prevalence. Several outbreaks of *C. striatum* MDR infections have been reported in varied countries from 1976 to 2020 [7]. An increase in the resistance rates to β-lactams, clindamycin, erythromycin, ciprofloxacin and gentamicin in *C. striatum* has been documented over the last 44 years. Currently, the few effective drugs available against MDR strains of this species are the glycopeptides, linezolid, quinupristin/dalfopristin, daptomycin, and tigecycline [8].

Reduced antibiotic susceptibility is also associated with the ability of *C. striatum* to form biofilms, which limits the action of the drugs on bacteria and promotes the exchange of resistance genes [9]. The biofilm is a microbial community enclosed in a self-produced polysaccharide matrix, adherent to biotic or abiotic surfaces. Several studies have demonstrated that bacteria resident in the biofilm readily acquire resistance determinants due to the cellular physical proximity in the polysaccharide matrix [10,11,12]. The emerging development of MDR bacteria in hospital settings underscores the need for searching new potential drugs to treat *C. striatum*-related infections.

Recently, drug-repurposing as an alternative approach to discover new potential antibiotics is attracting considerable interest [13]. Since these drugs are approved by the Food and Drug Administration (FDA), information on their pharmacological characteristics (chemical stability, toxicity, dosage, action kinetics, etc.) is readily available. This reduces the time and economic costs required to evaluate new therapeutic applications [14]. Several studies have demonstrated the antibacterial properties of anthelmintics against *Mycobacterium bovis*, *Mycobacterium tuberculosis*, *Helicobacter pylori*, *Enterococcus faecium* (*E. faecium*) and *Staphylococcus aureus* (*S. aureus*) [15,16,17,18]. Niclosamide is an anticestodal used in a wide range of hosts, which interferes with the parasite’s anaerobic metabolism [17]. Doramectin, moxidectin and selamectin act against heartworm larvae interrupting neurotransmission of the parasite, with consequent paralysis and death [14]. Contextually, this study aims to evaluate the effectiveness of doramectin, moxidectin, selamectin and niclosamide against *C. striatum* MDR isolates. To date, no studies have investigated the action of anthelmintic drugs against MDR *C. striatum*. Therefore, an antibacterial screening was conducted to envisage new therapeutic strategies against MDR *C. striatum* infection.

## 2. Results

The antimicrobial activity of niclosamide, doramectin, moxidectin and selamectin was investigated through disk diffusion, the plate microdilution method and biofilm degradation assays on 20 different clinical *C. striatum* isolates. The efficiency of the selected drugs was comparable among the different bacterial isolates under investigation; therefore, the data shown specifically refer to the *C. striatum* (S_1_) isolate and in-depth analyses, including a time-killing assay, scanning electron microscopy (SEM), and confocal laser scanning microscopy (CLSM), were also conducted exclusively on the *C. striatum* (S_1_) isolate.

### 2.1. Antibacterial Profile

**Figure 1:**
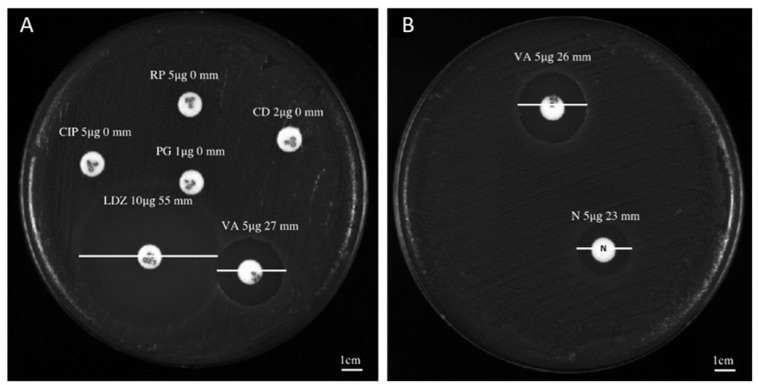
Antibacterial screening study of niclosamide and conventional antibiotics: (**A**) inhibition halo recorded for *C. striatum* (S_1_) after treatment with vancomycin (VA, 5 µg), linezolid (LDZ, 10 µg), ciprofloxacin (CIP, 5 µg), rifampicin (RP, 5 µg), benzylpenicillin (PG, 1 µg) and or clindamycin (CD, 2 µg); (**B**) diameter of the inhibition area exhibited by *C. striatum* (S_1_) after exposure with niclosamide (N, 5 µg).

The antibacterial screening of niclosamide, doramectin, moxidectin and selamectin against the collected *C. striatum* isolates was performed via Kirby–Bauer disk diffusion assays. The antibiogram and sites of origin showed a *C. striatum* MDR profile (Table 1). In detail, all isolates were resistant to ciprofloxacin (5 µg), rifampicin (5 µg), benzylpenicillin (1 µg) and clindamycin (2 µg), while they were sensitive to vancomycin (5 µg) and linezolid (10 µg), recording an inhibition diameter of 27 ± 1.0 and 55 ± 0.78 mm, respectively (Figure 1A). On the other hand, the inhibition area obtained after treatment with 5 μg niclosamide was 23 ± 1.2 mm (Figure 1B). No zone of inhibition was observed after treatment with doramectin, moxidectin and or selamectin (results not shown).

**Figure 2:**
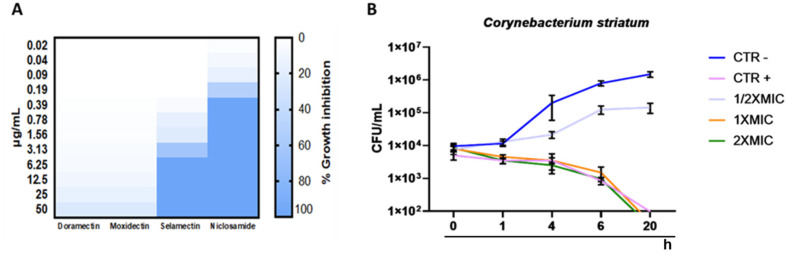
Antibacterial potential of anthelmintic drugs: (**A**) heatmap representation of bacterial inhibition after treatment of *C. striatum* S_1_ with doramectin, moxidectin, selamectin and niclosamide for 20 h; (**B**) time-kill kinetics of niclosamide against *C. striatum* cells. Negative control (CTRL-): untreated cells; CTRL+: bacteria treated with vancomycin (5 μg/mL); The data represent the mean ± standard deviation (SD).

**Figure 3:**
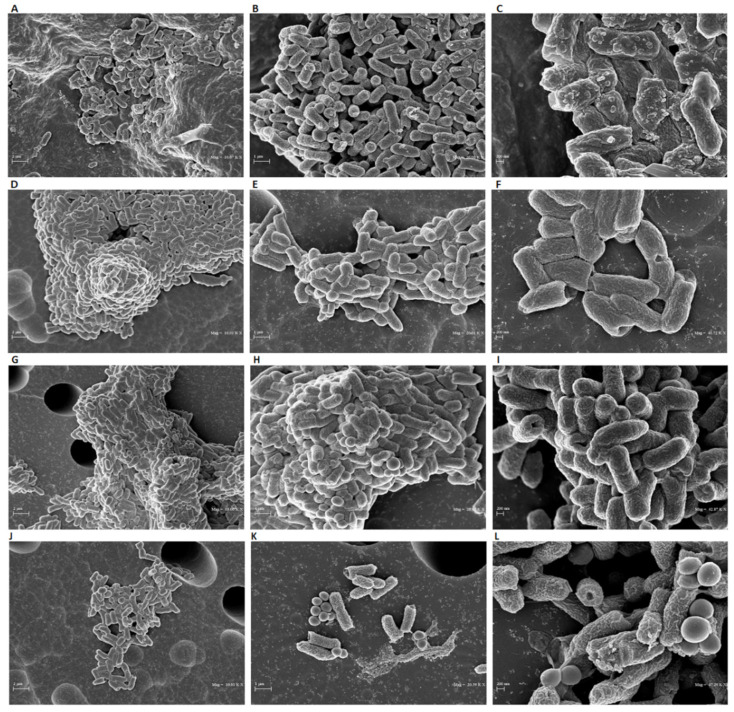
SEM analyses of *C. striatum* cells treated with niclosamide at 1 × MIC (**A**–**C**) and ½ × MIC (**D**–**F**). The negative CTRL was represented by bacteria treated with the solvent (**G**–**I**) and the positive CTRL was represented by the treatment with vancomycin (5 μg/mL) (**J**–**L**). The treatment was carried out for 20 h at 37 °C. The images were obtained (from left to right) at 10,000×, 20,000×, and 40,000× magnification.

To investigate the minimal inhibitory concentration (MIC), a dose-response curve of all isolates was determined following treatment with concentrations ranging from 0.02 to 50 μg/mL. Among the anthelminthic drugs, doramectin and moxidectin did not exhibit effective antibacterial activity in the concentration range tested, while selamectin recorded a MIC_90_ value of 6.25 μg/mL. Niclosamide represented the pharmacological agent with the best performing antibacterial activity, exhibiting a MIC_90_ value of 0.39 μg/mL (Figure 2A). Therefore, niclosamide was used as a drug of choice for extensive studies (Time-killing, SEM, CLSM). The kinetic action of niclosamide was evaluated by performing a time-killing curve analysis. Obtained data showed bacterial exponential growth over time. No significant alteration of microbial growth was reported following treatment with 0.19 μg/mL (½ × MIC) compared to the negative control. On the other hand, 4 h of exposure to doses of 0.39 (MIC) and 0.78 (2 × MIC) μg/mL induced a gradual reduction of the bacterial load, and no living cells were detected after 20 h (Figure 2B). These findings indicate an effective bactericidal activity of niclosamide. The effect of niclosamide on bacterial surface morphology was evaluated via SEM imaging. The data showed the appearance of morphological differences on the surface structures in response to the drug. The untreated bacteria showed intact and regular surfaces with pleomorphic morphology (coccoid, bacillary, or filamentous). Conversely, exposure to niclosamide caused structural changes resulting in the formation of bumps on the cell wall (Figure 3).

### 2.2. Estimation of the Biomass and Viability of Biofilm Cell

**Figure 4:**
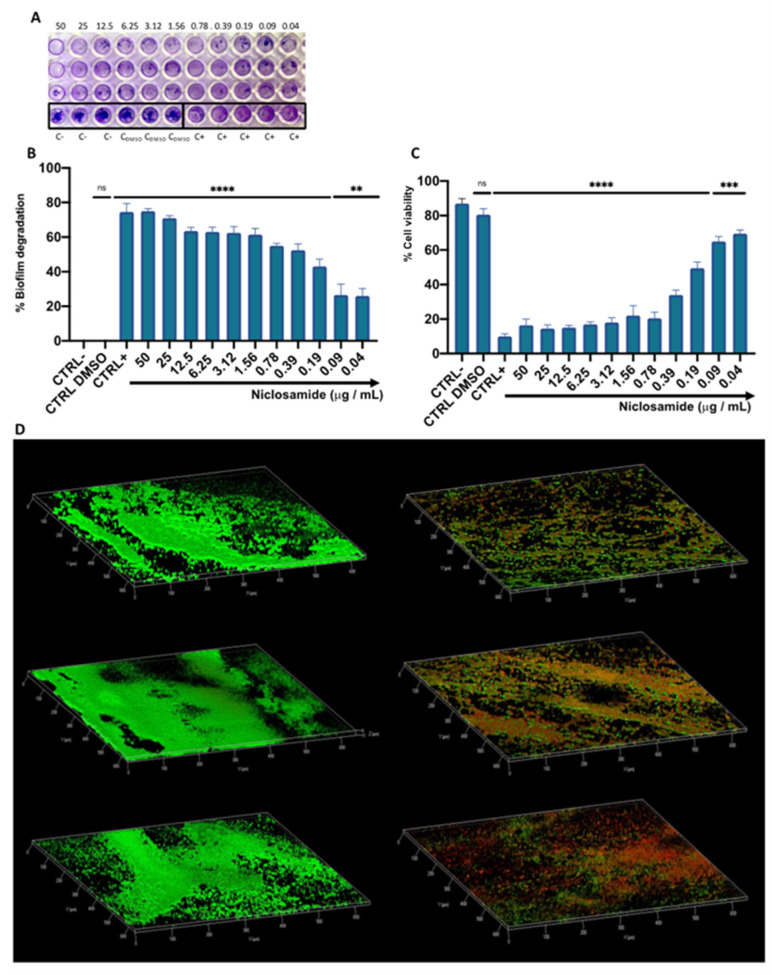
The effect of niclosamide on the preformed biofilm of *C. striatum*: (**A**,**B**) degradation of the biofilm matrix quantified by CV-staining; (**C**) Evaluation of the cell viability in the degraded biofilm; CTRL-: untreated biofilm; CTRL+: bacteria treated with vancomycin (20 μg/mL); CTRL DMSO: biofilm treated with the solvent used to dissolve the drug; The data represent the mean ± SD. Dunnett’s multiple comparisons test: **** *p* < 0.0001; *** *p* < 0.0005; ** *p* < 0.0096; ns *p* > 0.05. Ordinary one-way ANOVA: *p* < 0.0001, R^2^ = 0.9935; (**D**) confocal images of *C. striatum* biofilm treated with solvent (left) and niclosamide (right); red indicates dead cells, green points to live cells while yellow/orange is the result of the overlapping of dead and living cells; the images were acquisitions from three “randomly selected” areas.

The degradative effect on the mature biofilm was evaluated following treatment with niclosamide in a concentration range between 0.02 and 50 μg/mL. Biofilm biomass was quantified by a crystal violet (CV) assay (Figure 4A). After 20 h of treatment, the drug induced eradication of the biofilm matrix by 49 and 27% at 0.19 and 0.09 µg/mL, respectively, while the degradation rate was 55% in response to treatment with 0.39 µg/mL of the drug (Figure 4B). The viability of biofilm cells in the degraded biofilm was evaluated by 3-(4,5-dimethylthiazol-2-yl)-2,5-diphenyl tetrazolium bromide (MTT) assay. In agreement with the data obtained by CV-staining, the cell viability decreased in a dose-dependent manner by 16 to 70% in the tested concentration range. Specifically, at the doses of 0.19 and 0.39 μg/mL, the vital cellular load was 49 and 34%, respectively (Figure 4C). CLSM analysis supported the data obtained by CV and MTT assays (Figure 4D). The differential staining with SYTO9 (green fluorescence, live cells) and PI (red or yellow-red fluorescence, dead cells) showed that the treatment with niclosamide induced a conspicuous biofilm degradation and cellular death. Furthermore, the thickness of the biofilm was 10 µm in the untreated sample while it was reduced to 6 µm after treatment with 0.19 µg/mL niclosamide. These results demonstrate that the anthelmintic drug effectively reduced the biofilm biomass and impaired the viability of *C. striatum* matrix cells.

### 2.3. Cytotoxicity of Niclosamide

**Figure 5:**
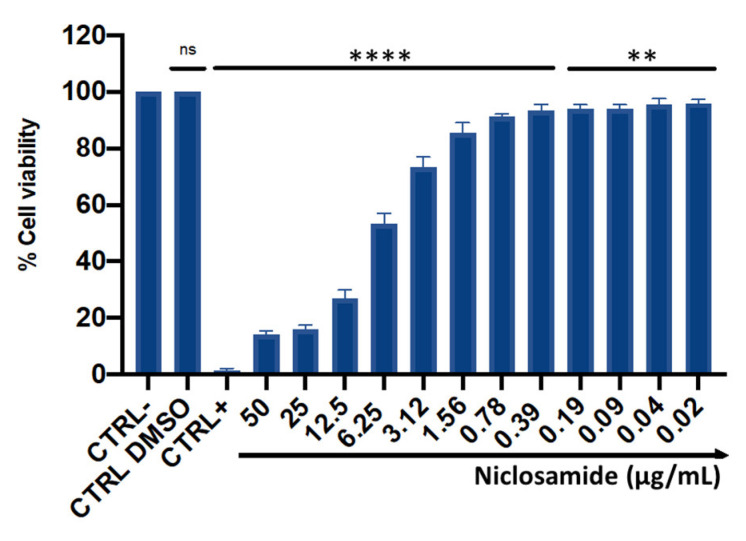
HaCaT cell viability after 24 h of treatment with niclosamide. CTRL-: untreated cells; CTRL+: dimethyl sulfoxide (DMSO) used at toxic concentration; CTRL DMSO: solvent used to dissolve the drug. The data represent the mean ± SD. Dunnett’s multiple comparisons test: **** *p* < 0.0001; ** *p* < 0.0096; ns *p* > 0.05. Ordinary one-way ANOVA: *p* < 0.0001, R^2^ = 0.9982.

The cytotoxic effect was evaluated using immortalized human keratinocyte (HaCaT) cells after 24 h of treatment with concentrations ranging between 0.02 and 50 μg/mL (Figure 5). The mortality rate increased in a dose-dependent manner with increasing concentrations, showing mortality of about 50% at 6.25 μg/mL. In the concentration range from 0.02 to 3.12 μg/mL, niclosamide did not induce substantial alterations in cell viability, recording a percentage of live cells of 80%. No cytotoxic effect was induced by the solvent used to dissolve the drug (DMSO), resulting in a cell viability of 98%. Furthermore, the 50% cytotoxic concentration (CC_50_) value, calculated from the dose-effect curves by non-linear regression analysis, was 2.56 μg/mL.

### 2.4. Bacterial Invasion and Intracellular Inhibition Assay

**Figure 6:**
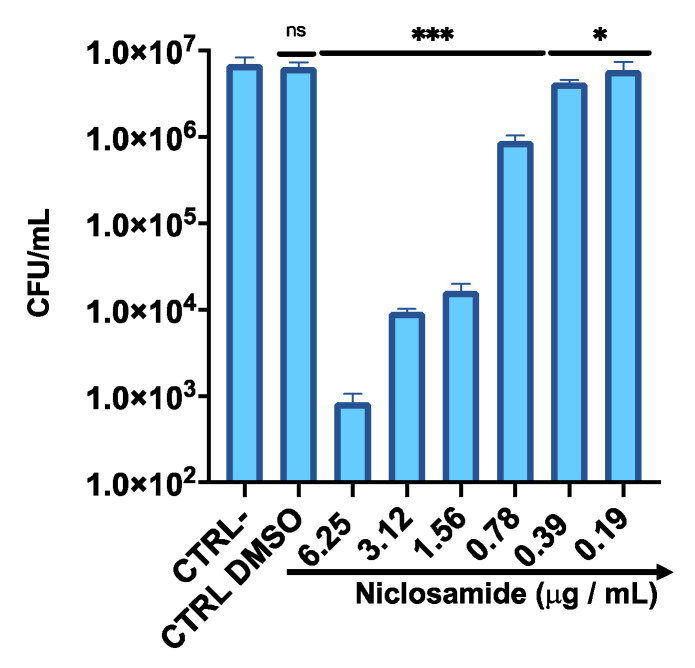
Intracellular survival of *C. striatum* in the HaCaT cell line after treatment with niclosamide. CTRL-: untreated cells; CTRL DMSO: cells treated with the solvent used to dissolve the drug. The data represent the mean ± SD. Dunnett’s multiple comparisons test: *** *p* < 0.0002; * *p* < 0.03. Ordinary one-way ANOVA: *p* < 0.0001, R^2^ = 0.9679.

To determine the antibacterial activity of niclosamide against the intracellular *C. striatum*, HaCaT cells were infected with bacteria (Figure 6). The infected cells were treated with niclosamide in a sub-cytotoxic concentration range, from 0.19 to 6.25 µg/mL, for 2 h post-invasion. The results indicated that a niclosamide concentration of 1.56 µg/mL was sufficient to inhibit the replication of *C. striatum* into HaCat cells by approximately 400 times, resulting in an intracellular load of 1.5 × 10^4^ CFU/mL relative to 7 × 10^6^ CFU/mL in untreated cells. On the other hand, no substantial inhibition of bacterial invasion was observed with lower concentrations of the drug.

## 3. Discussion

*C. striatum* is an emerging opportunistic pathogen commonly isolated from different biological districts, including wounds, bone biopsies and the bloodstream [19]. One of the main problems associated with *C. striatum* infections is the limited availability of appropriate drugs for antibacterial treatment. The resistance of *C. striatum* to a wide range of antimicrobial drugs highlights the need for novel therapeutic strategies [20]. A new promising approach to identify antimicrobial molecules from existing drugs is represented by drug-repurposing [21]. Anthelmintics are widely used and cost-effective drugs that could represent excellent candidates for reuse as antibacterial agents. Some anthelmintics have shown promising features for the treatment of bacterial infections. These compounds can be administered alone or in combination with other antibiotics to enhance their effectiveness [3].

Therefore, we performed an antibacterial screening of niclosamide, doramectin, moxidectin and selamectin against MDR *C. striatum* clinical isolates. Doramectin and moxidectin showed no antibacterial potential while selamectin and niclosamide negatively affected microbial growth. The choice to further investigate the action of niclosamide rather than selamectin was justified by two considerations: (i) niclosamide is an FDA approved anthelmintic drug for human use [14]; (ii) the MIC_90_ of selamectin (6.25 μg/mL) is 16 times higher compared to niclosamide (0.39 μg/mL). Therefore, further studies were electively focused to assess the activity of niclosamide against *C. striatum*.

The activity of niclosamide on mammalian cells was studied by evaluating the cytotoxicity on human keratinocytes; the drug induced a dose-dependent loss of cell viability, but the toxic effect (6.25 μg/mL) was observed at concentrations at least 16 times higher than the MIC-value against *C. striatum*. The niclosamide cytotoxicity profile has been extensively investigated, and in agreement with our results, several studies demonstrated low toxicity, whereby concentrations required to affect cell viability ranged from 5 to 8 μg/mL in human foreskin fibroblasts, sheep erythrocytes, human colon carcinoma, human colorectal adenocarcinoma and murine colorectal adenocarcinoma [14,22,23]. This overall evidence proved that niclosamide has a high degree of safety and a high therapeutic index.

The antibacterial potential against *C. striatum* isolates was evaluated using disk diffusion and broth microdilution methods. Niclosamide exhibited strong activity against all panels of the *C. striatum* isolates tested.

To date, no studies have evaluated niclosamide’s antibacterial potential against this emerging pathogen, while its effectiveness was assessed against other Gram-positive or Gram-negative bacterial species.

Niclosamide showed remarkable bactericidal activity against *C. striatum* isolates with a MIC_90_ value of 0.39 μg/mL. The action of niclosamide is associated with gross structural bacterial surface alterations, as demonstrated by SEM analysis. The images clearly showed that exposure to the drug induced a drastic reduction in the number of bacteria and morphological changes in the surviving cells through flaking of the wall and structural bruising. Domalaon et al. stressed a marked bactericidal efficacy of the drug on Gram-positive bacteria. Indeed, an MIC value ranging from 0.125 to 0.25 μg/mL was detected against *E. faecium* and *S. aureus*. In contrast, anMIC value greater than 64 μg/mL was obtained against *Klebsiella pneumoniae* (*K. pneumoniae*), *Acinetobacter baumanni (A. baumannii)*, *Pseudomonas aeruginosa* (*P. aeruginosa*) and *Enterobacter aerogenes*. [24]. Surprisingly, niclosamide completely suppressed the growth of *Helicobacter pylori* (*H. pylori*) with MIC values of 0.25 µg/mL [25]. Furthermore, niclosamide and colistin exerted synergic activity against colistin-sensitive (Col-S) and colistin-resistant (Col-R) *A. baumannii* and *K. pneumoniae*. In particular, the association of 1, 2 and 4 μM niclosamide and 0.5 μg/mL colistin decreased the MIC of colistin against all Col-S and Col-R isolates [26]. The synergistic effect could be due to the alteration of the surface negative charge in the outer membrane of these bacteria. The different activity of the drug could be attributed to the structural and molecular differences between the different bacterial species. For example, the resistance of *P. aeruginosa* to tetracycline, chloramphenicol and norfloxacin is attributed to the presence of efflux pumps on the outer membrane, absent in Gram-positive bacteria, against which these drugs are active [27]. Recent studies have reported the effect of niclosamide on proton motive force (PMF) [28,29]. All bacteria require PMF for the synthesis of ATP by the ATPase enzyme complex [28]. Tharmalingam et al. proved that niclosamide exposure causes alteration of the membrane potential, disrupting the *H. pylori* PMF [25]. Similarly, Copp et al. showed that colistin-niclosamide’s co-administration increased the efficacy of the anthelmintic drug against *E. coli* PMF [29]. Dissipation of the PMF leads to the impairment of the inner membrane integrity and rapid cell death [30]. Due to the bactericidal action of niclosamide, we hypothesize that exposure to the drug interrupts bacterial PMF, causing the loss of cytoplasmic membrane integrity, the formation of bumps on the external surface and consequent cell death. Further investigations will be necessary to verify the proposed mode of action.

Corynebacterium is also reported as a non-obligated intracellular pathogen, allowing the formation of an intracellular reservoir leading to chronic infection [17]. Therefore, we investigated the activity of niclosamide against *C. striatum* through a gentamicin protection test. Notably, the drug at 0.78 and 0.39 μg/mL reduced the infection rate in human keratinocytes by about twofold and 10-fold, respectively.

In the second set of experiments, we investigated the action of niclosamide against mature *C. striatum* biofilm. The CV and MTT assays indicated that the drug had a strong degradative activity on the biofilm matrix. In detail, the biomass degradation exceeded 50% and 40% in response to treatment with 1 × MIC and ½ × MIC of the drug, reaching the deeper layers of the mature biofilm and altering the viability of the resident matrix cells. CLSM data confirmed the degradation potential of niclosamide, showing a reduction in the viability of biofilm cells after drug exposure. A study conducted by Imperi et al. showed that niclosamide inhibited the quorum detection signal (QS) of *P. aeruginosa* by modulating the transcription of QS-dependent regulon target genes. Consequently, suppression of motility, inhibition of the virulence factors elastase, pyocyanin, and rhamnolipids, and the reduction of biofilm formation were reported [5].

In conclusion, given that antimicrobial resistance is compelling the scientific community to seek new therapeutic options, the attempt at repurposing drugs could prove pivotal for the rapid identification of novel compounds to manage the risks associated with the development and spread of MDR bacteria in the nosocomial environment. Our findings demonstrate that niclosamide represents a safe therapeutic agent with strong antibacterial and antibiofilm activity against *C. striatum*, which is commonly resistant to conventional antibiotics. Further investigations will be needed to elucidate the mechanism of action and to verify in appropriate in vivo models the action of this drug in the treatment of *C. striatum* infections.

## 4. Materials and Methods

### 4.1. Preparation of the Compound

Niclosamide, doramectin, moxidectin and selamectin were purchased by Sigma-Aldrich (St. Louis, MI, USA). All compounds were dissolved in dimethyl sulfoxide (DMSO) at the final concentration of 1 mg/mL and stored at −20 °C until use (Table 2).

### 4.2. Collection and Characterization of Bacterial Isolates

In this study, 20 isolates were collected at the “Luigi Vanvitelli” University Hospital of Campania (Naples, Italy) in the period between January and February 2021. Each isolate was plated on a Columbia agar plate supplemented with 5% sheep blood (Oxoid, Cheshire, UK) and incubated overnight (ON) at 37 °C. Thereafter, bacterial identification was performed by matrix-assisted laser desorption ionization-time of flight mass spectrometry (MALDI-TOF MS) (Bruker Dal-tonics, Bremen, Germany). Briefly, one colony was plotted on a metal target plate and coated with 1 μL of α-cyano-4 hydroxycinnamic acid matrix solution for 2 min at room temperature (RT). The mass spectrometer, through laser desorption/ionization, automatically generated the MALDI-TOF spectrum related to the bacterium. The obtained spectra were imported into MALDI BioTyper 3.0 software (Bruker Dal-tonics, Bremen, Germany) and evaluated by standard pattern matching concerning the spectra in the database. A score greater than 2 was attributed to the confident identification of the species [31].

Antimicrobial susceptibility of *C. striatum* isolates was performed by a Kirby–Bauer Disk Diffusion test, according to the guidelines of the National Committee on Clinical Laboratory Standards (NCCLS) [32]. For each isolate, a fresh colony was inoculated in saline solution up to a turbidity of 0.5 McFarland. The bacterial suspension was homogeneously seeded with a sterile swab on Mueller Hinton Agar with 5% Sheep Blood (MHF) (Oxoid, Cheshire, UK). Vancomycin (5 μg), linezolid (10 μg), ciprofloxacin (5 μg), rifampicin (5 μg), benzylpenicillin (1 μg) and clindamycin (2 μg) disks (Thermo Fisher Scientific, Waltham, MA, USA) were placed on the plate. Plates were incubated at 37 °C ON and the diameter of inhibition was measured. The interpretation criteria (S/R) were evaluated following the guidelines of the European Committee for Antibiotic Susceptibility Testing (EUCAST) [33].

### 4.3. Kirby–Bauer Disk Diffusion Test

The initial screening for testing the antibacterial activity against *C. striatum* isolates was performed through the Kirby–Bauer Disk Diffusion assay, as described above. An empty disk was soaked with 5 µg of niclosamide, doramectin, moxidectin and selamectin, respectively, placed on MHF-agar plate and incubated ON at 37 °C. Vancomycin (5 μg) was used as a CTRL+. After incubation, the diameters of the inhibition areas were measured.

### 4.4. Determination of the Minimum Inhibitory Concentration

The antibacterial potential of the selected drugs was assessed through the plate microdilution assay, according to the Clinical and Laboratory Standards Institute (CLSI) guidelines [34]. A colony of *C. striatum* was inoculated in Brain Heart Infusion (BHI) broth at 37 °C under orbital shaking (180 rpm) ON. The following day, 300 μL of inoculum was added to 15 mL of fresh medium and the bacterial suspension was incubated up to the middle log-phase [optical density (OD) at 600 nm of 0.5]. The inoculum was diluted in BHI-broth to obtain a bacterial suspension of 1 × 10^6^ CFU/mL, suitable for the assay. A volume of 50 μL of the inoculum was added to each well, obtaining a final density of 5 × 10^5^ CFU/well. Meanwhile, niclosamide, doramectin, moxidectin and selamectin were serially diluted in phosphate-buffered saline (PBS) 1× in a concentration range of 0.02 to 50 μg/mL. Vancomycin (5 μg/mL), solvent drug, and untreated bacteria were used as CTRL+, solvent CTRL, and CTRL-. Thereafter, the plates were incubated at 37 °C and the growth rate was evaluated after 20 h using a microplate reader (Tecan, Männedorf, Switzerland).

### 4.5. Time Killing Assay

Bacterial growth was monitored over time in response to the treatment of niclosamide. Concentrations of ½ × MIC (0.19 μg/mL), 1 × MIC (0.39 μg/mL) and 2 × MIC (0.78 μg/mL) were set up in BHI broth at a final volume of 2 mL/tube. Untreated bacteria and vancomycin-treated bacteria were used as CTRL- and CTRL+. A bacterial suspension of 1 × 10^6^ CFU/mL was added to each tube and incubated at 37 °C with orbital shaking (180 rmp). A volume of 100 μL at time intervals of 0, 1, 3, 6 and 20 h was serially diluted in BHI broth, plated on BHI agar and incubated at 37 °C ON. Afterwards, the colonies were counted to define the CFU/mL.

### 4.6. SEM Analyses

The morphological modifications induced by niclosamide were investigated by SEM. The bacterial (*C. striatum* S_1_) load of 5 × 10^5^ CFU/mL was used for niclosamide treatment at concentrations of 0.39 and 0.19 μg/mL. Untreated and vancomycin-treated bacteria represented, respectively, the CTRL+ and CTRL-. Thereafter, the bacteria were fixed in 4% paraformaldehyde and dehydrated with cold solutions of ethanol at increasing concentrations (30, 50, 70, 90, 95 and 100% *v/v*). The images were acquired using the ZEISS Supra 40 (EHT = 5.00 kV, WD = 22 mm, the detector in the lens) (Berlin, Germany) [35].

### 4.7. Biofilm Degradation and Viability

The ability of niclosamide to degrade biofilm was assessed by CV colorimetric assay, while the viability of biofilm cells was evaluated via MTT colorimetric assay. For both tests, the overnight-grown bacterial inoculum was diluted to a concentration of 2 × 10^8^ CFU/mL in BHI supplemented with 1% glucose. A volume of 100 μL of the bacterial suspension was added to each well of the 96-well plate and incubated at 37 °C for 20 h to ensure biofilm formation. Thereafter, the planktonic cells were removed by washing twice with PBS 1× and the mature biofilm was treated with niclosamide from 0.04 to 50 μg/mL for 20 h at 37 °C. Vancomycin (20 μg/mL) and untreated bacteria were used as CTRL+ and CTRL-. After treatment, the medium was discarded and the biofilm was washed with 1× PBS. To quantify the biofilm biomass, the matrix was stained with 0.1% CV (Thermo Fisher Scientific, Waltham, MA, USA) for 40 min at RT. The excess CV was removed by washing with PBS 1× and the stained biofilm was solubilized with 100% DMSO. The viability of biofilm cells was assessed, incubating treated and untreated biofilm with 5 mg/mL of MTT solution for 3 h at 37 °C. Later, MTT was removed and the formed formazan crystals were solubilized with 100% DMSO. Absorbance values at 570 nm were measured using a microplate reader for both assays.

### 4.8. CLSM Analysis

CLSM was used to observe the niclosamide effect on the preformed biofilm of *C. striatum*. The biofilm was produced on a Nunc^®^ Lab-Tek^®^ II chamber slide (Sigma-Aldrich, St. Louis, MI, USA) and degradation was observed at a concentration of 0.19 μg/mL. The bacteria were stained in the LIVE/DEAD BacLight bacterial viability kit (Molecular Probes, Eugene, OR, USA) following the manufacturer’s instructions. Images were captured using an inverted LSM 710 confocal laser scanning microscope (Zeiss, Oberkochen, Germany) and analyzed by CLSM Z-Stack analysis: the software was set up to acquire an az series of 1 µm thick optical sections collected sequentially along the *z*-axis on the whole biofilm samples and three-dimensional reconstructions were generated.

### 4.9. Cell Culture and Cytotoxicity Assays

HaCaT cells were used to evaluate the cytotoxicity of niclosamide by MTT assay. The cells were grown in DMEM (Thermo Fisher Scientific, Waltham, MA, USA), supplemented with 1% penicillin-streptomycin and 10% fetal bovine serum (Thermo Fisher Scientific, Waltham, MA, USA), at 37 °C with 5% CO_2_ in a humid environment. A density of 2 × 10^4^ cells/well was seeded in 96-well plates and incubated for 20 h. A concentration range between 0.04 and 50 μg/mL of niclosamide was used for cell treatment. Untreated cells, cells treated with drug solvent and 100% DMSO were used as negative, solvent and positive controls, respectively. After 20 h of treatment, 100 µL of MTT solution was added to each well for 3 h at 37 °C. The solubilization of the formazan crystals was achieved by adding 100 µL of 100% DMSO and the viability rate was recorded at OD at 570 nm, using a microplate reader (Tecan, Männedorf, Switzerland) [36,37].

### 4.10. Intracellular Activity of Niclosamide

The cell internalization assay was performed to evaluate the role of niclosamide in the invasion of *C. striatum* (S_1_) on the HaCaT cell line. Cells were seeded in 24-well plates at a density of 1.5 × 10^5^ cells/well for 24 h. After 2 h of starvation, cell monolayers were infected at a multiplicity of infection (MOI) of 1 for 3 h at 37 °C with 5% CO_2_. Subsequently, the medium was removed and cells were washed twice with 1× PBS to remove planktonic and weakly attached bacteria. Gentamicin (100 μg/mL) was added for 2 h and cell monolayers were washed twice in 1× PBS. Niclosamide in the concentration range of 0.19 to 6.25 μg/mL was chosen for HaCaT treatment for 2 h at 37 °C with 5% CO_2_. After washing, the cells were lysed with cold 0.1% Triton-X for 5 min to free intracellular bacteria. Serial dilutions of the cell lysates were plated on Columbia agar with sheep blood and incubated ON at 37 °C. Colonies were counted to determine CFU/mL.

### 4.11. Statistical Analysis

All experiment were performed in three technical and biological replicates. The data from each experiment represent the mean (SD) of three biological replicates and were processed using GraphPad Prism ver. 8.2.1 for macOS (GraphPad Software, San Diego, CA, USA, www.graphpad.com, accessed on 27 February 2020). The statistical analysis included the One-way ANOVA followed by Dunnett’s multiple comparison tests. The results were considered statistically significant with a *p*-value ≤ 0.05.

## Figures and Tables

**Table 1:** Isolation sites of isolates the and inhibition zone of vancomycin (VA, 5 µg), linezolid (LDZ, 10 µg), ciprofloxacin (CIP, 5 µg), rifampicin (RP, 5 µg), clindamycin (CD, 2 µg), benzylpenicillin (PG, 1 µg), niclosamide (NP, 5 µg), doramectin (DO, 5 µg), selamectin (SE, 5 µg) and moxidectin (MO, 5 µg) against *C. striatum* strains. ND, not detected.

Bacterium Characteristics		Zone Diameter Breakpoints [mm—Sensitive (S)/Resistant (R)]									
Isolates	Biological Material of Origin	VA (5 μg)	LZD (10 μg)	CIP (5 μg)	RP (5 μg)	CD (2 μg)	PG (5 μg)	NP (5 μg)	DO (5 μg)	SE (5 μg)	MO (5 μg)
*C. striatum* S_1_	Bronchial aspirate	27 (S)	53 (S)	0 (R)	0 (R)	0 (R)	0 (R)	23	ND	ND	ND
*C. striatum* S_2_	Wound swab	23 (S)	50 (S)	0 (R)	0 (R)	0 (R)	0 (R)	22	ND	ND	ND
*C. striatum* S_3_	Urine	26 (S)	55 (S)	0 (R)	0 (R)	0 (R)	0 (R)	24	ND	ND	ND
*C. striatum* S_4_	Swab ulcer	27 (S)	53 (S)	0 (R)	0 (R)	0 (R)	0 (R)	22	ND	ND	ND
*C. striatum* S_5_	Peripheral venous blood	25 (S)	55 (S)	0 (R)	0 (R)	0 (R)	0 (R)	23	ND	ND	ND
*C. striatum* S_6_	Swab ulcer	25 (S)	55 (S)	0 (R)	0 (R)	0 (R)	0 (R)	24	ND	ND	ND
*C. striatum* S_7_	Swab ulcer	26 (S)	53 (S)	0 (R)	0 (R)	0 (R)	0 (R)	23	ND	ND	ND
*C. striatum* S_8_	Swab ulcer	22 (S)	56 (S)	0 (R)	0 (R)	0 (R)	0 (R)	23	ND	ND	ND
*C. striatum* S_9_	Pus	27 (S)	52 (S)	0 (R)	0 (R)	0 (R)	0 (R)	22	ND	ND	ND
*C. striatum* S_10_	Peripheral venous blood	27 (S)	54 (S)	0 (R)	0 (R)	0 (R)	0 (R)	24	ND	ND	ND
*C. striatum* S_11_	Swab ulcer	25 (S)	56 (S)	0 (R)	0 (R)	0 (R)	0 (R)	24	ND	ND	ND
*C. striatum* S_12_	Central venous blood catheter	27 (S)	56 (S)	0 (R)	0 (R)	0 (R)	0 (R)	24	ND	ND	ND
*C. striatum* S_13_	Swab ulcer	23 (S)	53 (S)	0 (R)	0 (R)	0 (R)	0 (R)	22	ND	ND	ND
*C. striatum* S_14_	Sputum	27 (S)	50 (S)	0 (R)	0 (R)	0 (R)	0 (R)	23	ND	ND	ND
*C. striatum* S_15_	Swab ulcer	26 (S)	54 (S)	0 (R)	0 (R)	0 (R)	0 (R)	21	ND	ND	ND
*C. striatum* S_16_	Swab ulcer	24 (S)	53 (S)	0 (R)	0 (R)	0 (R)	0 (R)	23	ND	ND	ND
*C. striatum* S_17_	Throat swab	25 (S)	54 (S)	0 (R)	0 (R)	0 (R)	0 (R)	23	ND	ND	ND
*C. striatum* S_18_	Bronchial aspirate	25 (S)	54 (S)	0 (R)	0 (R)	0 (R)	0 (R)	24	ND	ND	ND
*C. striatum* S_19_	Urinary catheter	27 (S)	53 (S)	0 (R)	0 (R)	0 (R)	0 (R)	22	ND	ND	ND
*C. striatum* S_20_	Bone biopsy	24 (S)	55 (S)	0 (R)	0 (R)	0 (R)	0 (R)	23	ND	ND	ND

**Table 2:** Chemical structure and molecular properties of moxidectin, selamectin, niclosamide and doramectin.

	Moxidectin	Selamectin
Chemical structure	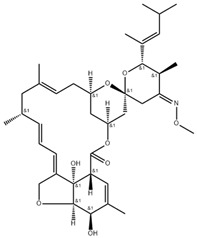	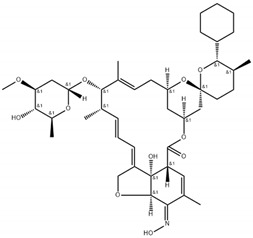
Molecular formula	C_37_H_53_NO8	C_43_H_63_NO_11_
Molecular weight	639.82 g/mol	769.97 g/mol
Appearance	white crystals	yellow crystals
Density	1.2 g/cm^3^	1.35 g/cm^3^
Melting point	145–154 °C	N/A
Boiling point	790 °C	917.0 °C
Solubility in water	0.51 mg/L	0.43 mg/L
	**Niclosamide**	**Doramectin**
Chemical structure	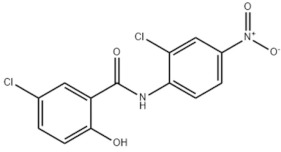	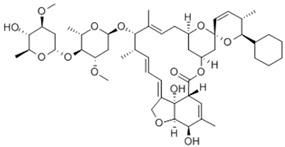
Molecular formula	C_13_H_8_Cl_2_N_2_O_4_	C_50_H_74_O_14_
Molecular weight	327.12 g/mol	899.11 g/mol
Appearance	yellow crystals	white crystals
Density	1.6 g/cm^3^	1.25 g/cm^3^
Melting point	225–230 °C	116–119 °C
Boiling point	424.5 °C	967.4 °C
Solubility in water	13.32 mg/L	0.025 mg/L
